# A Low-FODMAP Diet Improves the Global Symptoms and Bowel Habits of Adult IBS Patients: A Systematic Review and Meta-Analysis

**DOI:** 10.3389/fnut.2021.683191

**Published:** 2021-08-19

**Authors:** Jinsheng Wang, Pengcheng Yang, Lei Zhang, Xiaohua Hou

**Affiliations:** Department of Gastroenterology, Tongji Medical College, Union Hospital, Huazhong University of Science and Technology, Wuhan, China

**Keywords:** FODMAP, diet, irritable bowel syndrome, quality of life, meta-analysis, HADS

## Abstract

**Background:** A low-fermentable oligo-, di-, monosaccharides, and polyols (FODMAP) diet has been reported to be associated with improving the symptoms of irritable bowel syndrome (IBS); however, its efficacy as evaluated by different studies remains controversial.

**Objective:** A systematic review and meta-analysis of randomized controlled trials (RCTs) were conducted to explore the efficacy of a low-FODMAP diet (LFD) in alleviating the symptoms of IBS.

**Methods:** A search of the literature for RCTs that assessed the efficacy of an LFD in treating IBS patients was conducted using the electronic databases PubMed, Embase, Cochrane Central Register of Controlled Trials, and Web of Science. The searches in each database were conducted from the inception of the database to February 2021. Two independent reviewers screened citations and a third reviewer resolved disagreements. Two independent reviewers also performed eligibility assessments and data extraction. The RCTs that evaluated LFDs vs. a normal IBS or usual diet and assessed changes of IBS symptoms were included in the search. Data were synthesized as the relative risk of global symptoms improvement, mean difference of IBS Severity Scoring System (IBS-SSS) score, sub-items of IBS-SSS irritable bowel syndrome-related quality of life (IBS-QOL), hospital anxiety and depression scale (HADS), stool consistency/frequency, and body mass index (BMI) using a random effects model. The risk of bias was assessed using Risk of Bias Tool 2 (RoB 2). The bias of publication was assessed based on Egger's regression analysis. The quality of evidence was assessed using the Grading of Recommendations Assessment, Development and Evaluation (GRADE) methodology.

**Results:** A total of 2,768 citations were identified. After full-text screening, a total of 10 studies were eligible for the systematic review and were subsequently used to compare an LFD with various control interventions in 511 participants. An LFD was associated with the improvement of global symptoms [*n* = 420; Risk Ratio (RR) = 1.54; 95% Confidence Interval (CI) 1.18 to 2; *I*^2^ = 38%], improvement of stool consistency [*n* = 434; Mean difference (MD) = −0.25; 95% CI −0.44 to −0.06; *I*^2^= 19%), and a reduction trend of stool frequency (*n* = 434; MD = −0.28; 95% CI −0.57 to 0.01; *I*^2^ = 68%) compared with control interventions. There was no statistically significant change in IBS-QOL (*n* = 484; MD = 2.77; 95% CI −2 to 7.55; *I*^2^ = 62%), anxiety score (*n* = 150; MD = −0.45; 95% CI −3.38 to 2.49; *I*^2^ = 86%), depression score (*n* = 150; MD = −0.05; 95% CI −2.5 to 2.4; *I*^2^ = 88%), and BMI (*n* = 110; MD = −0.22; 95% CI −1.89 to 1.45; *I*^2^ = 14%). The overall quality of the data was “moderate” for “global improvement of IBS symptom,” “stool consistency,” “stool consistency for IBS with diarrhea (IBS-D),” and “stool frequency for IBS-D,” and “low” or “very low” for other outcomes according to GRADE criteria.

**Conclusion:** An LFD is effective in reducing the global symptoms and improving the bowel habits of adult IBS patients. The efficacy for IBS-D patients can also be more pronounced.

**Systematic Review Registration:** CRD42021235843.

## Introduction

Irritable bowel syndrome is one of the most prevalent chronic gastrointestinal diseases, with a prevalence of ~7–21% (1, 2). In some Western countries, the prevalence of irritable bowel syndrome (IBS) is around twice as high in females than in males, which may be higher in Asian countries (1). The diagnosis of IBS is based on the association of recurrent abdominal pain with altered bowel habits, namely, diarrhea and/or constipation, in the absence of organic diseases, such as inflammatory bowel disease or colon cancer (2). IBS is usually categorized into subtypes according to predominant bowel habits: IBS with constipation (IBS-C), IBS with diarrhea (IBS-D), mixed IBS (IBS-M), or unsubtyped IBS (IBS-U) (1–3). Irritable bowel syndrome has been conceptualized as a brain–gut disorder (4), which is also associated with poor quality of life, impaired social function (5), and psychological-psychiatric conditions, such as anxiety and depression (6–8). Medications that improve diarrhea (e.g., loperamide, probiotics) or constipation (e.g., fiber supplements, laxatives) are used as the first-line IBS therapies to improve altered bowel habits but offer little benefit for abdominal pain, bloating, and psychosocial problems (1, 2). Up to 70% of IBS patients report that symptom onset or exacerbation are associated with certain food, such as milk and milk products, wheat products, caffeine, cabbage, onion, peas, beans, hot spices, and fried and smoked food (3, 9–11). Some IBS patients tend to avoid certain food items and try gluten-free or lactose-free diets to prevent the onset of their symptoms (12, 13). However, these avoidances of food may make them susceptible to long-term nutritional deficiencies and low body weight (14).

Restricting food with highly fermentable oligo-, di-, monosaccharides, and polyols (FODMAPs), which can trigger and/or exacerbate IBS symptoms, may contribute to managing IBS symptoms according to a growing body of clinical trials (15–19). Examples of FODMAPs include fructose, lactose, sugar alcohols (sorbitol, maltitol, mannitol, xylitol, and isomalt), fructans, and galactans, which are widely presented in a large range of food, such as wheat, rye, vegetables, fruits, and legumes (20).

Fermentable oligo-, di-, monosaccharides, and polyols might exacerbate IBS symptoms through various mechanisms, such as increasing small intestinal water volume, colonic gas production and intestinal motility (21). A series of high-quality randomized controlled trials (RCTs) have been conducted to assess the efficacy of a low-FODMAP diets (LFDs) in IBS (15, 17–19, 21–26). However, these resulted in controversial conclusions.

Five recent meta-analyses (27–31) have been performed on this topic. However, none of them paid enough attention to the efficacy of LFD on stool output and psychological or psychiatric conditions in IBS patients. Therefore, this study was aimed to conduct an updated and more comprehensive meta-analysis of RCTs, evaluate the effects of LFD therapy for IBS patients to improve their symptoms and IBS-QOL, stool consistency and frequency score, anxiety and depression score based on hospital Anxiety and Depression Scale (HADS), and body mass index (BMI).

## Methods

This systematic review and meta-analysis was conducted in accordance with the Preferred Reporting Items for Systematic Reviews and Meta-Analysis (PRISMA) statement (32) and was registered on the International Prospective Register of Systematic Reviews (PROSPERO) (registration number CRD42021235843).

### Search Strategy

The search of the literature for RCTs that assessed the efficiency of an LFD in treating with IBS was conducted using the electronic databases PubMed, Embase, Cochrane Central Register of Controlled Trials, and Web of Science. The searches in each database were conducted from the inception of the databases to February 2021. Search terms included “‘irritable bowel syndrome' OR ‘IBS”' AND “‘fermentable oligosaccharides, disaccharides, monosaccharides, and polyols' OR ‘fodmap,' OR ‘fermentable oligo-, di- and monosaccharides and polyols.”' No language restrictions were used in the search process.

### Study Selection

The inclusion criteria were presented as the following: (1) randomized controlled trials (including cross-over trials); (2) participants aged ≥ 18 years, (3) an objective basis for diagnosis (Rome I, II, III, or IV); (4) comparing LFD with a placebo diet or a usual diet; (5) outcomes including global improvement in IBS symptoms, IBS-QOL, HADS, stool consistency/frequency, or BMI; (6) the duration of therapy ≥ 3 weeks. The exclusion criteria were presented as the following: (1) non-randomized controlled trials, cohort studies, retrospective studies, or case reports, (2) participants aged <18 years, (3) participants suffered from other digestive disorders, such as inflammatory bowel disease, (4) participants in the experimental group received multiple interventions at the same time. Two independent reviewers (Wang JS and Yang PC) performed the screening of the citations and a third reviewer (Zhang L) resolved disagreements.

### Outcome Assessment

The primary outcome was assessed according to the global improvement in IBS symptoms. Secondary outcomes included IBS-QOL, stool consistency/frequency, HADS, and BMI.

### Data Extraction

Two independent reviewers (WJ and YP) performed the data abstraction for this study. Data extracted included data on the year of publication, country of origin, design of the study, clinically meaningful improvement standard, duration of therapy, IBS criteria, IBS subtype involved, the comparator intervention, and outcomes. Risk ratio of symptom improvement was abstracted as an intention-to-treat analysis, and the dropouts would be treated in the groups to which they had been initially randomized. The mean difference of the IBS Severity Scoring System (IBS-SSS) score, sub-items of IBS-SSS (including “pain intensity,” “pain frequency,” “abdominal distension,” “dissatisfaction of bowel habit,” and “interference on life in general”), IBS-QOL score, HADS score, stool consistency and frequency score, and BMI were assessed. Disagreements were resolved by a third reviewer (ZL).

### Assessment of Risk of Bias and GRADE Methodology

The risk of bias assessment was performed by two independent reviewers (WJ and YP) using the Cochrane Risk of Bias Tool with Review Manager (RevMan) (Version 5.3, Cochrane Collaboration). Each study was evaluated based on the reporting of randomization, allocation, blinding, and outcome assessment and reporting. Data was analyzed to assess the quality of evidence according to GRADE (Grading of Recommendations Assessment, Development and Evaluation) methodology using the GRADEPro Guideline Development Tool (GDT) (33).

### Data Synthesis and Statistical Analysis

Data analysis was performed using RevMan 5 (Version 5.3, Cochrane Collaboration). The risk ratio (RR) was calculated with 95% confidence intervals (CIs) of symptoms improving, mean difference (MD) with 95% confidence intervals of IBS-SSS score, IBS-QOL score, HADS score, stool consistency and frequency score, and BMI in the IBS with LFD group compared with control. Data were pooled with a random effects model. Heterogeneity was evaluated with the *I*^2^ statistic, with >50% considered to be significant heterogeneity. Forest plots were used with RRs or MDs for primary or secondary outcomes. The 153Publication bias was assessed based on Egger's regression analysis (using the Stata 16 software). The reasons for heterogeneity were explored using subgroup analyses based on the definition of clinically meaningful improvement for IBS global symptoms, type of control intervention, duration of treatment, and subtype of IBS.

## Results

### Search Results and Study Selection

**Figure 1:**
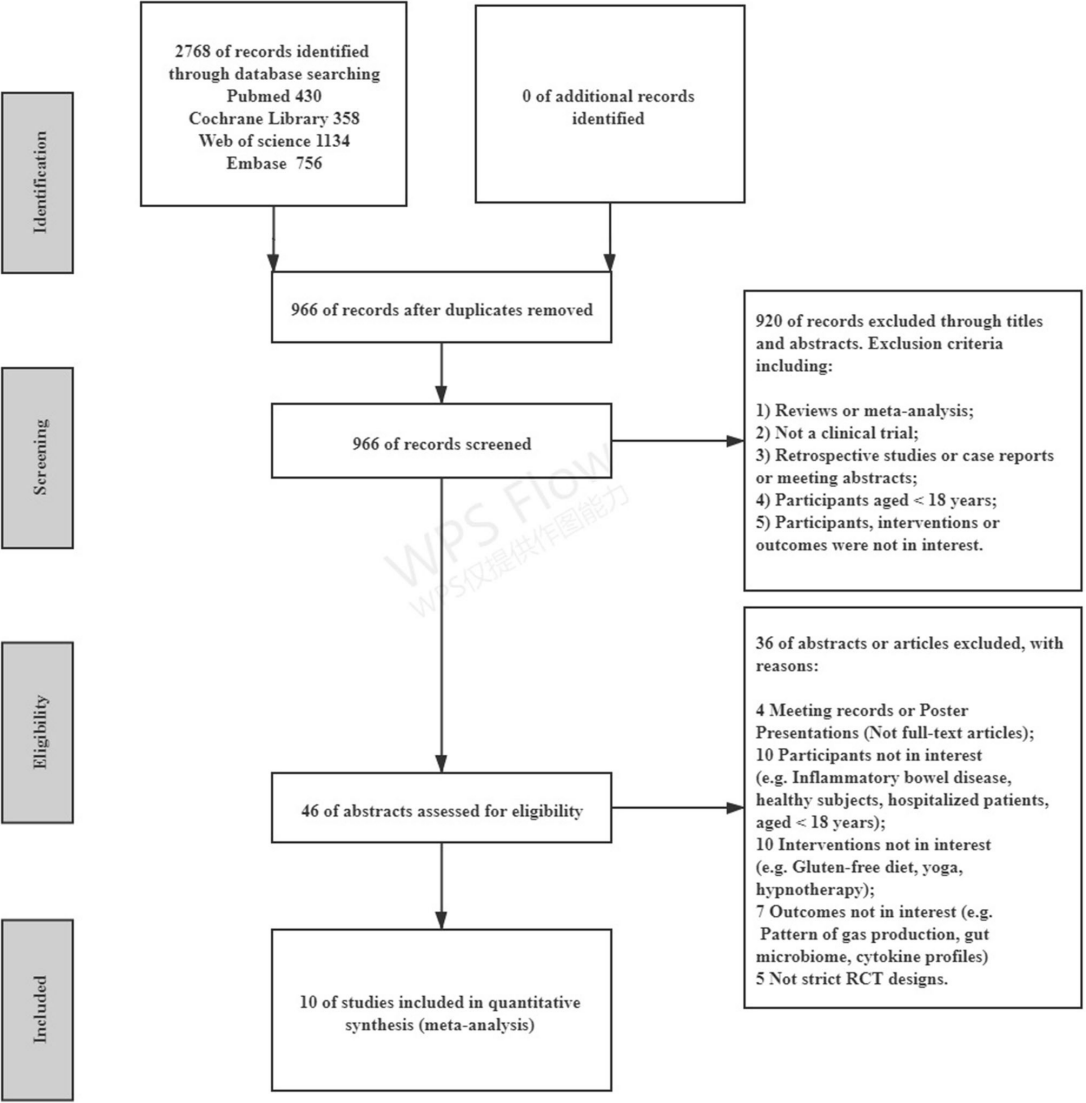
Preferred reporting items for systematic reviews and meta-analyses (PRISMA) flow diagram of the included studies. RCT, randomized controlled trial.

The literature search identified 2,768 citations through electrical databases, and 46 studies underwent full manuscript review. After full-text screening, 36 articles were excluded for different reasons, leaving a total of 10 studies that were eligible for the systematic review, comparing an LFD with control diets (including the traditional IBS diet, high-FODMAP diet, or usual diet) in 511 participants (Figure 1). A summary of the trial characteristics is given in Table 1.

**Table 1:** Baseline characteristics of the studies included in the meta-analysis.

**References**	**Design**	**Duration**	**IBS definition**	**IBS type**	**Age range or mean age (SD)/year**	**Female/total**	**Intervention**	**Participants (LFD/ND)**	**Drops (LFD/ND)**	**Clinically meaningful improvement**	**Symptom assessment**
Böhn et al. (22)Sweden	Multi-center parallel single-blind RCT	4 weeks	ROME III	IBS-DIBS-CIBS-MIBS-U	43 (16)	56/67	Traditional IBS diet vs. LFD	33/34	5/3	A reduction in IBS-SSS ≥ 50	IBS-SSS Stool consistency/ frequency BMI HADS
Halmos et al. (21)Australia	Single-blind cross-over RCT	3 weeks	ROME III	IBS-DIBS-CIBS-MIBS-U	23–60	21/30	Typical Australian diet vs. LFD	30/24	5/2	A reduction in VAS ≥ 10 mm	100-mm (VAS)
McIntosh et al. (18)Canada	Single-blind parallel RCT	3 weeks	ROME III	IBS-DIBS-CIBS-MIBS-U	18–52	32/37	High FODMAP diet vs. LFD	18/19	5/2	A reduction in IBS-SSS ≥ 50	IBS-SSS
Staudacher et al. (24)the UK	RCT	4 weeks	ROME III	IBS patients with bloating and/or diarrhea as major IBS symptom	LFD:35.2 (11.4) ND:35.0 (8.7)	23/35	Habitual diet vs. LFD	19/16	1/2	Answer “yes” to “Were your symptoms adequately controlled over the previous week?”	Global symptom question; Stool consistency/ frequency
Staudacher et al. (25)the UK	Multi-center 2 ×2 factorial RCT	4 weeks	ROME III	IBS-DIBS-MIBS-U	LFD:36 (11) ND:33 (12)	70/104	Sham diet vs. LFD	51/53	2/1	Answer “yes” to “Did you have adequate relief of your symptoms over the past 7 days?”	“Adequate symptom relief” question; IBS-SSS; IBS-QOL; Stool consistency/ frequency
Wilson et al. (26)the UK	Double-blind 3-arm RCT	4 weeks	ROME III	IBS-DIBS-CIBS-MIBS-U	LFD:38.9 (10.0) ND:30.3 (9.8)	25/45	Sham diet vs. LFD	21/21	4/3	Answer “yes” to “Over the past 7 days, do you feel that you have had adequate relief of your IBS symptoms?”	“Adequate symptom relief” question; IBS-SSS; IBS-QOL; Stool consistency/ frequency
Zahedi et al. (19)Iran	Single-blind RCT	6 weeks	ROME III	IBS-D	LFD:37.60 (11.9) ND:37.43 (13.27)	51/101	GDA vs. LFD	50/51	2/3	–	IBS-SSS; IBS-QOL; Stool consistency/ frequency; HADS
Eswaran et al. (23)America	Single-center single-blind RCT	4 weeks	ROME III	IBS-D	LFD:41.6 (14.7) ND:43.8 (15.2)	65/92	mNICE vs. LFD	45/39	8/9	–	IBS-QOL; HADS
Eswaran et al. (15)America	Single-center single-blind RCT	4 weeks	ROME III	IBS-D	LFD:41.6 (14.7) ND:43.8 (15.2)	65/92	mNICE vs. LFD	45/39	8/9	Answer “yes” to “In regard to all your IBS symptoms, as compared with the way you felt before you started the diet, have you, in the past seven days, had adequate relief of your IBS symptoms?”	“Adequate symptom relief” question; Stool consistency/ frequency
Laatikainen et al. (17)Finland	Double blind cross-over RCT	4 weeks	ROME III	IBS-DIBS-MIBS-U	42.9 (21–64)	73/80	Regular rye bread vs. Low-FODMAP rye bread	80/80	4/6	–	IBS-SSS IBS-QOL

*RCT, randomized controlled trial; BMI, body mass index; FODMAP, fermentable oligo-di-mono-saccharides and polyols; LFD, low fermentable oligo-di-mono-saccharides and polyols diet; GDA, general dietary advice; IBS-D, irritable bowel syndrome with diarrhea; IBS-M, mixed stool pattern irritable bowel syndrome; IBS-C, constipation predominant irritable bowel syndrome; IBS-U, unclassified irritable bowel syndrome; VAS, visual analog scale; HADS, hospital anxiety and depression scale; IBS-SSS, irritable bowel syndrome-severity symptom scale; IBS-QOL, irritable bowel syndrome related quality of life; mNICE, modified diet recommended by the National Institute for Health and Care Excellence; vs., versus*.

### Global Improvement of Symptoms

**Figure 2:**
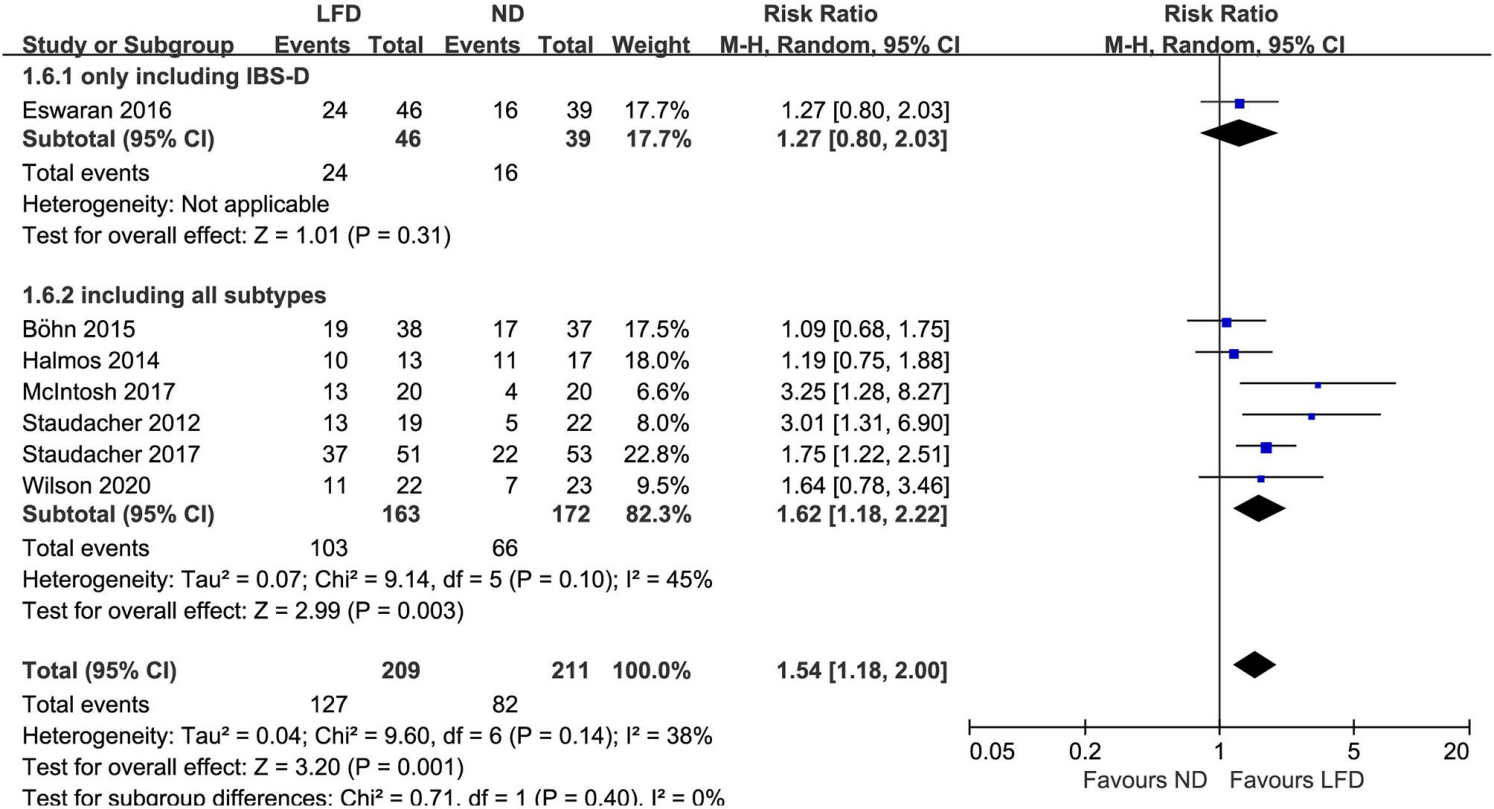
Pooled relative risk for the improvement of irritable bowel syndrome (IBS) global symptoms. LFD, low-FODMAP diet; ND, normal diet.

**Figure 3:**
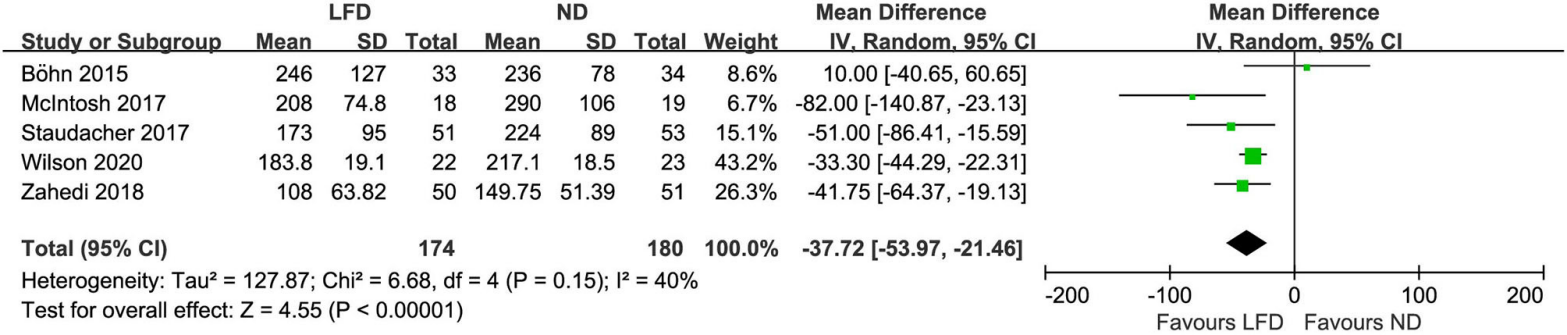
Pooled mean difference for the improvement of IBS global symptoms. LFD, low-FODMAP diet; ND, normal diet.

Seven studies reported the global improvement of symptoms with different clinically meaningful improvement definitions as dichotomous outcomes (Figure 2), where an LFD was associated with an improvement of global symptoms in IBS patients compared with controls (*n* = 420; RR = 1.54; 95% CI 1.18–2; *I*^2^ = 38%). Five studies assessed global symptom changes using IBS-SSS as continuous variables, showing that an LFD was associated with a reduction in total IBS-SSS score (*n* = 354; MD = −37.72; 95% CI −53.97 to −21.46; *I*^2^ = 40%) (Figure 3), pain intensity (*n* = 354; MD = −11.27; 95% CI −16.32 to −6.23; *I*^2^ = 47%) (Supplementary Figure 1), pain frequency (*n* = 354; MD = −9.11; 95% CI −16.26 to −1.96; *I*^2^ = 73%) (Supplementary Figure 2), interference on life in general (*n* = 354; MD = −11.58; 95% CI −13.92 to −9.24; *I*^2^ = 0%) (Supplementary Figure 3), and dissatisfaction of bowel habit (*n* = 354; MD = −8.95; 95% CI −12.6 to −5.31; *I*^2^ = 26%) (Supplementary Figure 4), but with no statistically significant effect on abdominal distension (*n* = 354; MD = −4.82; 95% CI −10.75 to 1.11; *I*^2^ = 57%) (Supplementary Figure 5).

### Stool Output

**Figure 4:**
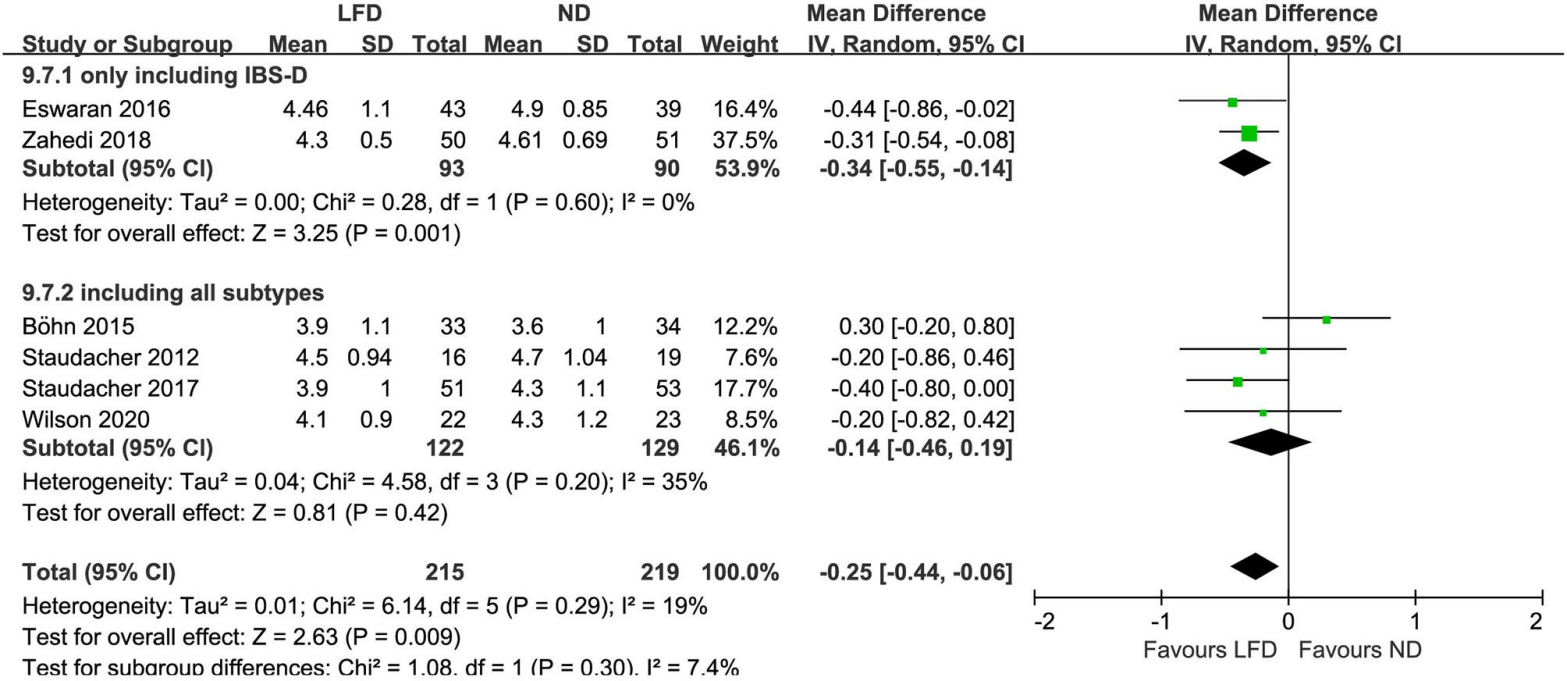
Pooled mean difference for stool consistency based on the Bristol Stool Form Scale. LFD, low-FODMAP diet; ND, normal diet.

**Figure 5:**
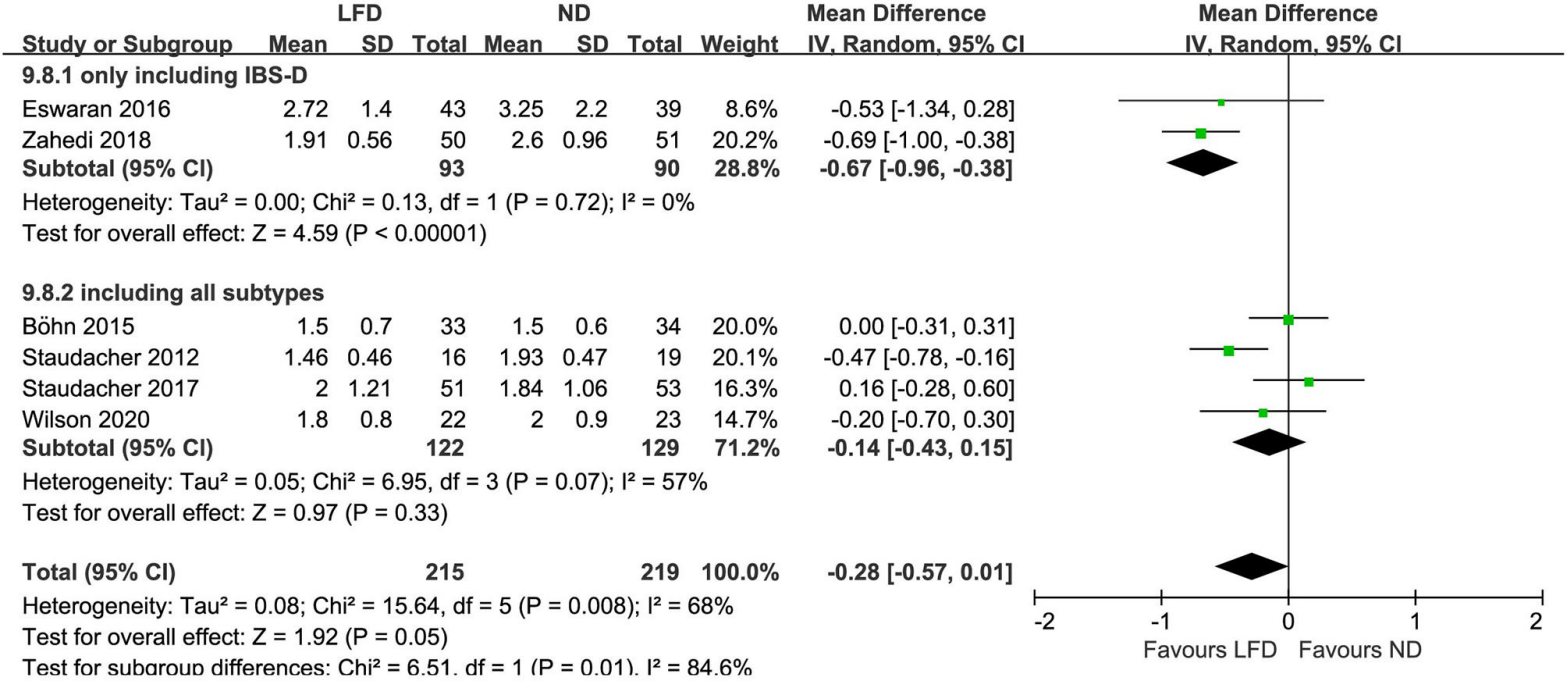
Pooled mean difference for stool frequency (per day). LFD, low-FODMAP diet; ND, normal diet.

Six studies reported the improvement of stool output in IBS patients due to an LFD. An LFD also showed significant effects on stool consistency scores (*n* = 434; MD = –.25; 95% CI −0.44 to −0.06; *I*^2^ = 19%) (Figure 4), and a trend of reduced stool frequency per day (*n* = 434; MD = −0.28; 95% CI −0.57 to 0.01; *I*^2^ = 68%) (Figure 5) compared with control interventions. Interestingly, the improvement of stool output in IBS-D patients seemed to be more sensitive to an LFD according to subgroup analysis: stool consistency score (*n* = 183; MD = −0.34; 95% CI −0.55 to −0.14; *I*^2^ = 0%) (Figure 4) and stool frequency (*n* = 183; MD = −0.67; 95% CI −0.96 to −0.38; *I*^2^ = 0%) (Figure 5).

### IBS-QOL

**Figure 6:**
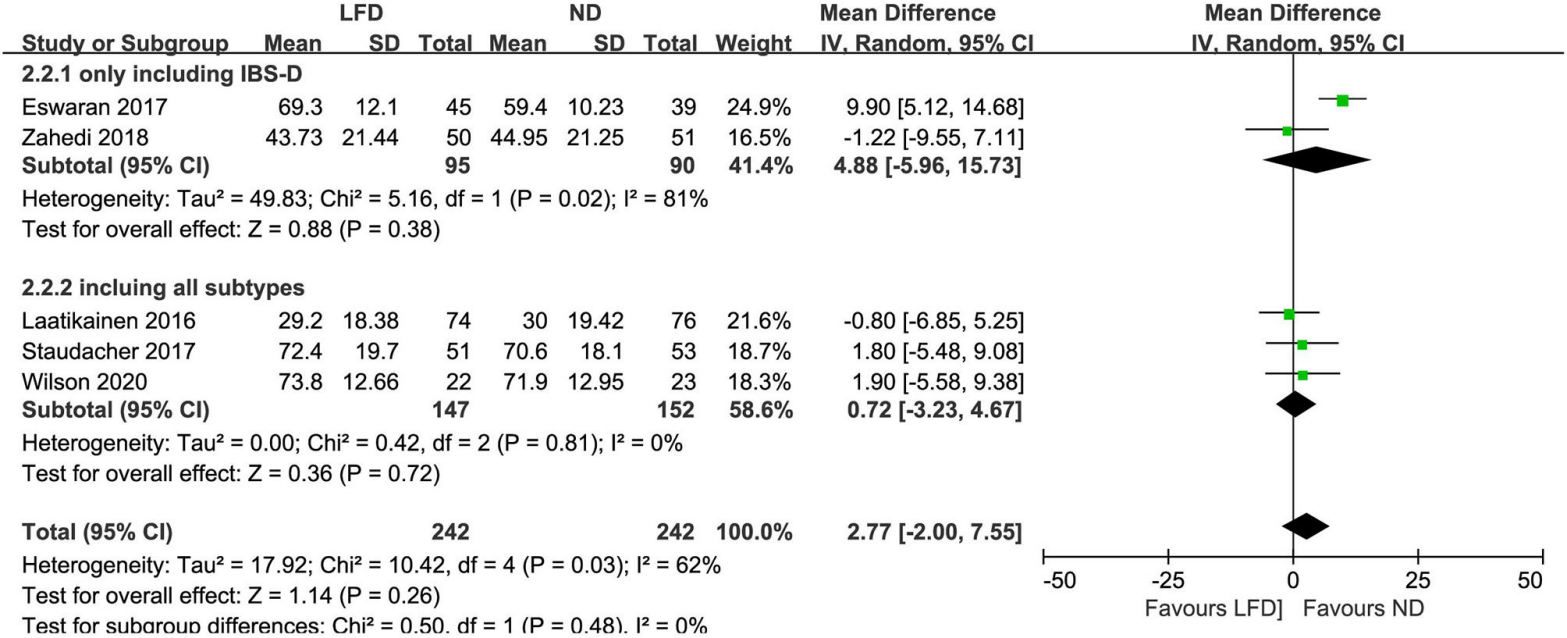
Pooled mean difference for irritable bowel syndrome-related quality of life (IBS-QOL). LFD, low-FODMAP diet; ND, normal diet.

The irritable bowel syndrome-related quality of life score was analyzed using the synthesis from five studies, showing no significant changes (*n* = 484; MD = 2.77; 95% CI −2 to 7.55; *I*^2^ = 62%). Subgroup analysis based on IBS subtype showed no statistical difference between subgroups (*p* = 0.48) (Figure 6).

### HADS

**Figure 7:**
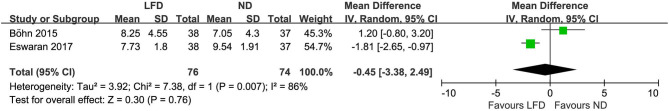
Pooled mean difference for anxiety score based on the hospital anxiety and depression scale. LFD, low-FODMAP diet; ND, normal diet.

**Figure 8:**
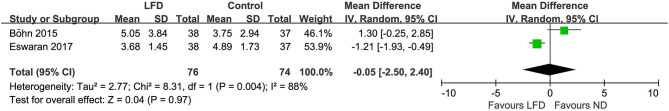
Pooled mean difference for depression score based on the hospital anxiety and depression scale. LFD, low-FODMAP diet; ND, normal diet.

Two studies reported HADS, however, both showed no difference between low-FODMAP groups and controls: anxiety score (*n* = 150; MD = −0.45; 95% CI −3.38 to 2.49; *I*^2^ = 86%) (Figure 7) and depression score (*n* =150; MD = −0.05; 95% CI −2.5 to 2.4; *I*^2^ = 88%) (Figure 8).

### BMI

**Figure 9:**
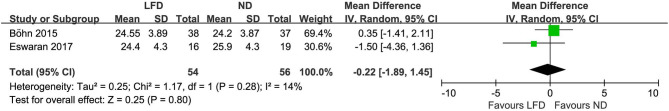
Pooled mean difference for body mass index (BMI). LFD, low-FODMAP diet; ND, normal diet.

Only two studies reported the effect of LFD on BMI changes, but showed no statistical difference (*n* = 110; MD = −0.22; 95% CI −1.89 to 1.45; *I*^2^ = 14%) (Figure 9).

### Risk of Bias and GRADE

**Figure 10:**
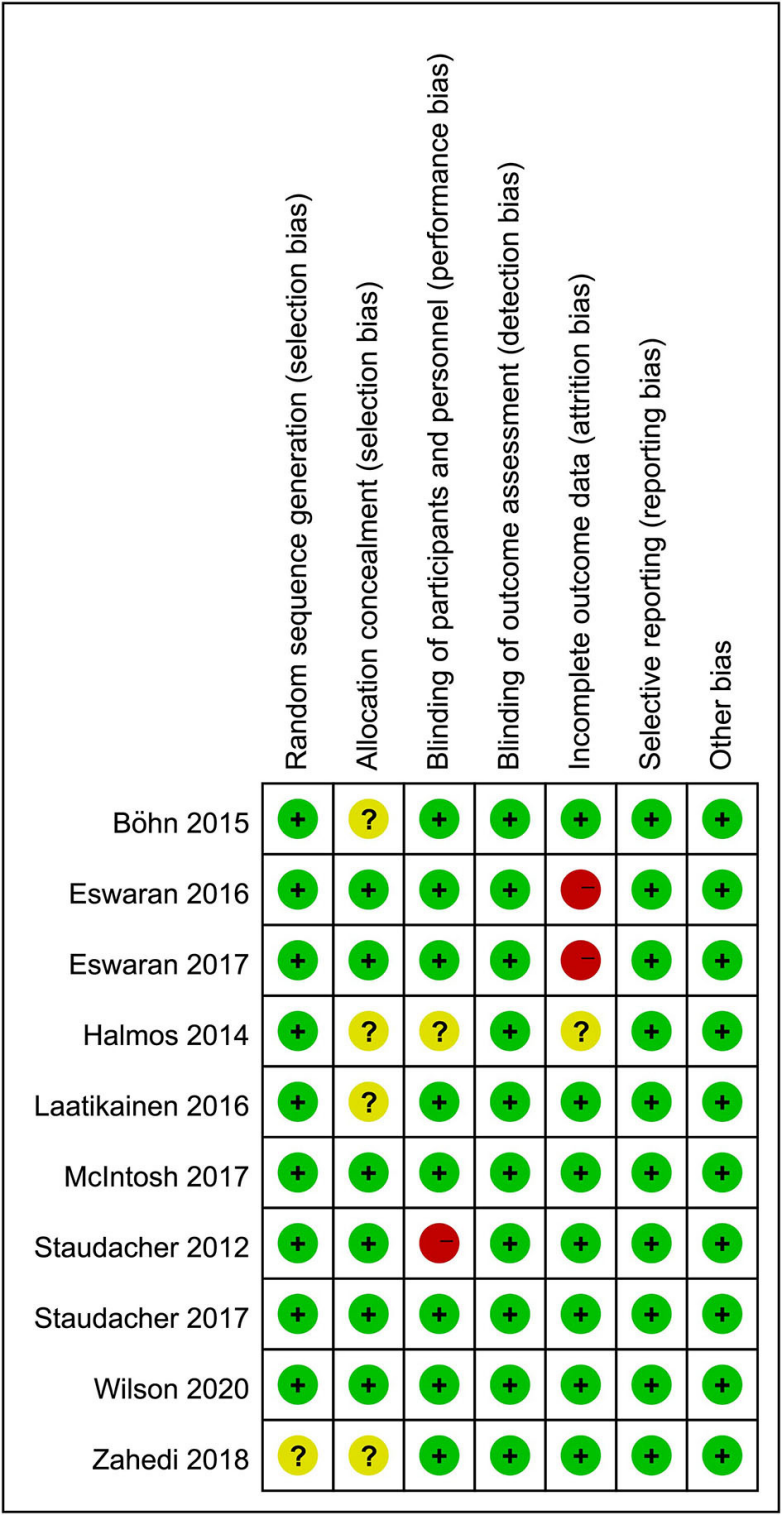
Summary of risk of bias.

The overall risk of bias is relatively low as shown in Figure 10. A summary of the quality of evidence according to GRADE for the included RCTs is given in Table 2.

**Table 2:** Grading of recommended assessment, development and evaluation (GRADE) summary of findings.

**Certainty assessment**							**Summary of findings**				
**Participants (studies) follow up**	**Risk of bias**	**Inconsistency**	**Indirectness**	**Imprecision**	**Publication bias**	**Overall certainty of evidence**	**Study event rates (%)**		**Relative effect (95% CI)**	**Anticipated absolute effects**	
							**With normal diet**	**With LFD**		**Risk with normal diet**	**Risk difference with LFD**
**Global improvement of IBS symptom**											
420 (7 RCTs)	Serious	Not serious	Not serious	Not serious	None	□□□○ MODERATE	82/211 (38.9%)	127/209 (60.8%)	RR 1.58 (1.29–1.93)	389 per 1,000	225 more per 1,000 (from 113 more to 361 more)
**Stool consistency**											
434 (6 RCTs)	Serious	Not serious	Not serious	Not serious	None	□□□○ MODERATE	219	215	–	The mean stool consistency was 4.41	MD 0.27 lower (0.43 lower to 0.11 lower)
**Stool frequency**											
434 (6 RCTs)	Serious	Not serious	Not serious	Serious	None	□□○○ LOW	219	215	–	The mean stool frequency was 2.24	MD 0.28 lower (0.57 lower to 0.01 higher)
**Stool consistency for IBS-D**											
183 (2 RCTs)	Not serious	Not serious	Not serious	Serious	None	□□□○ MODERATE	90	93	–	The mean stool consistency for IBS-D was 4.74	MD 0.34 lower (0.55 lower to 0.14 lower)
**Stool frequency for IBS-D**											
183 (2 RCTs)	Not serious	Not serious	Not serious	Serious	None	□□□○ MODERATE	90	93	–	The mean stool frequency for IBS-D was 2.88	MD 0.67 lower (0.96 lower to 0.38 lower)
**IBS related quality of life**											
555 (6 RCTs)	Serious	Serious	Not serious	Serious	None	□○○○ VERY LOW	279	276	–	The mean IBS related quality of life was 51.59	MD 2.66 higher (1.42 lower to 6.74 higher)
**Anxiety score**											
150 (2 RCTs)	Serious	Serious	Not serious	Serious	None	□○○○ VERY LOW	74	76	–	The mean anxiety score was 8.30	MD 0.45 lower (3.38 lower to 2.49 higher)
**Depression score**											
150 (2 RCTs)	Serious	Very serious	Not serious	Serious	None	□○○○ VERY LOW	74	76	–	The mean depression score was 4.32	MD 0.05 lower (2.5 lower to 2.4 higher)
**BMI**											
110 (2 RCTs)	Serious	Not serious	Not serious	Serious	None	□□○○ LOW	56	54	–	The mean BMI was 24.78	MD 0.16 lower (1.65 lower to 1.34 higher)

*CI, Confidence interval; RR, Risk ratio; MD, Mean difference*.

### Publication Bias

There was no evidence of publication bias based on Egger's regression analysis: global improvement of symptoms (*p* = 0.0765); IBS-SSS (*p* = 0.1558); pain intensity (*p* = 0.7638); pain frequency (*p* = 0.7686); abdominal distension (*p* = 0.7689); dissatisfaction of bowel habit (*p* = 0.1871); interference on life in general (*p* = 0.0785); IBS-QOL (*p* = 0.1086); stool consistency (*p* = 0.4353); stool frequency (*p* = 0.9699).

### Subgroup Analysis

Subgroup analysis of the outcomes (except “HADS” and “BMI” because only 2 RCTs were included for each outcome) was conducted based on “treatment duration,” “FODMAP level in the control diet,” “definition of clinically meaningful improvement,” and “IBS subtype.” Results are shown in Supplementary Table 1.

## Discussion

This updating meta-analysis included 10 high-quality RCT studies involving 511 participants according to the above criteria. The study aimed to provide clinicians with evidence-based data proving that an LFD alleviates symptoms in patients with IBS effectively. More research data were attempted to be extracted from existing studies to explore the effects of an LFD on the overall symptoms, stool output, IBS-QOL, anxiety and depression, and BMI of IBS patients. The study found that an LFD significantly reduced the global symptoms of patients with IBS and improved their stool output, especially for those with IBS-D, and that the quality of evidence was moderate. However, LFDs had no statistically significant effects on IBS-QOL, anxiety and depression score, and BMI in patients with IBS, while the quality of evidence was low or very low. The reasons for the low level of evidence quality mainly include inappropriate blinding methods, large heterogeneity, and a limited number of studies. Even though some potential limitations and concerns of an LFD have been raised, such as nutritional adequacy, cost, difficulty in teaching, learning, and continuing, most of the limitations (20, 34). In conclusion, based on the evidence presented in this meta-analysis, adult IBS patients, especially those with IBS-D, are recommended to try an LFD with professional advice from health care professionals.

Global symptom improvement was treated as the primary outcome in this systematic review. Seven studies (15, 18, 21, 22, 24–26) evaluated the effectiveness of an LFD in improving the overall symptoms of IBS with dichotomous variables, using different clinically meaningful improvement criteria. Meanwhile, there were 5 RCTs (18, 19, 22, 25, 26) that used IBS-SSS to assess the IBS global symptoms with continuous variables, which also supported this conclusion. According to the results, over 60% (127/209) of IBS patients in the LFD group experienced significant relief, which seems to be an acceptable result; whereas, GRADE (35) would ideally require 300 responders to be classified as robust. More large-sample studies are needed to provide reliable evidence in the future. However, it is a challenge to conduct a high-quality RCT on this subject due to a lack of support from the pharmaceutical industry and funding agencies (27). Three previous meta-analyses used “mean difference” or “standardized mean difference” based on IBS-SSS as their effect sizes (30, 31, 36). These two kinds of effect sizes can only reflect the effect of an LFD on the IBS population, but cannot evaluate individual differences. At the same time, a statistically significant mean difference may not be clinically significant. For example, a 50-point reduction in IBS-SSS is generally considered to reflect a clinically meaningful improvement (22, 37). Thus, risk ratio (RR) was chosen to evaluate the difference between LFD and control diets, trying to make the results more clinically meaningful and easier to understand. In addition, comparing responder rates between trials is difficult because of the different responder definitions that were used (22). A 50-point reduction of IBS-SSS in two trials (18, 22) and a 10-point reduction in the visual analog scale (VAS) in one trial (21) were considered to reflect a clinically meaningful improvement. On the other hand, patients who felt adequate relief of their IBS symptoms were seen as responders in another four papers (15, 24–26). The existing scales for assessing the severity of IBS symptoms are not uniform, and it makes no sense to directly pool these scores together for meta-analysis. If the continuous variables of scores from a scale can be transformed into a dichotomous variable according to an appropriate “responding criteria,” the results of these clinical trials can be directly compared despite the different scales. Moreover, dropout is inevitable in clinical research and the reasons should be clarified. For instance, in patients who were intolerant of the intervention: the data of these results should be attributed to treatment failure rather than simple data loss in an intention-to-treat analysis.

According to ROME III (2, 38) and IV (4, 39) criteria, IBS is diagnosed on the basis of recurrent abdominal pain related to defecation or in association with a change in stool frequency or form. Thus, the effect of LFDs on altered bowel habits in IBS patients is an important aspect to evaluate. To our knowledge, this is the first meta-analysis that included stool output as a crucial outcome on this subject, which has not been demonstrated by previous meta-analyses (27–31). Stool consistency generally refers to the rheology or viscosity of the stool, which is largely determined by stool water content (40, 41). Gastrointestinal water absorption is limited by rapid intestinal transit limits, causing loose or liquid stools (42). It can be measured as a finite number of categories by the Bristol Stool Form Scale (BSFS), which is the most widely used criteria (43, 44). According to our study, pooled data from six RCTs showed a moderate improvement in the stool output of IBS patients following an LFD, which was consistent with a previous meta-analysis study (only containing three RCTs for this outcome) (28). Interestingly, patients with IBS-D (however, only 93 IBS-D patients were included) seemed to benefit more from an LFD, probably obtaining a greater improvement in stool output than other IBS subtypes according to the subgroup analysis. The results mentioned above indicate that an LFD may contribute to reduced stool water content, increase stool hardness, and further reduce stool frequency effectively. Nevertheless, according to this theory, constipation in IBS-C patients would not be improved by an LFD and may even be worsened. However, more research is needed in the future to confirm this.

Irritable bowel syndrome affects the quality of life negatively (44–46), to the same degree as organic gastrointestinal disorders like Crohn's disease (47). This imposes a substantial burden on patients and employers (45, 46), which suggests a significant unmet need for effective therapies to treat the symptoms of IBS and alleviate the considerable societal and patient burden associated with this condition. The IBS-QOL, validated in 1998 by Patrick et al. (48) is utilized as a conceptually valid self-administered questionnaire with highly reproducible results for assessing the perceived quality of life for individuals with IBS (48). Meaningful clinical improvement is seen by a rise in IBS-QOL score > 14 (48). Six RCTs involved the evaluation of the effects of an LFD on this term with relatively high heterogeneity. Sensitivity analysis showed that the greatest heterogeneity among the studies came from Eswaran et al. (23). When this study was excluded, the heterogeneity index *I*^2^ decreased from 62 to 0%. However, the final result was still not statistically significant, suggesting that no clinical improvement in this term occurred after an LFD intervention in IBS patients. Consistent results were observed in the subgroup analysis based on IBS subtype. It is important to note that restrictive diets can sometimes be stressful for patients with chronic diseases. Any effort to eliminate more food or impose further dietary restrictions might hamper the adherence rate, produce opposite results, and have a negative effect on the quality of life in patients with IBS (49). In the LFD group, in particular, available dietary choices were restricted to a great degree, reducing long-term adherence (20, 33). Ooi et al. (50) and Halmos (51) noted that extensive or inappropriate use of the LFD could have a negative impact on the health of patients. On the other hand, the duration of most LFD trials was limited (<8 weeks) and could not ensure long-term efficacy comparable to the drug trials (52). An additional period may be necessary for clinically significant improvement in quality of life for IBS patients to manifest following an LFD.

Major psychosocial problems have been reported to be observed in 50–60% of IBS patients (6). Three pieces of meta-analyses showed that levels of anxiety and depression were significantly higher in IBS patients compared with healthy controls (6–8). Meanwhile, the prevalence rates of anxiety and depression symptoms in IBS patients are near 40 and 30%, respectively (6). It is not difficult to accept that chronic IBS symptoms can have a destabilizing impact on quality of life and be associated with stress, work impairment, and further aggravation of mental disorders. However, there was no significant difference in the anxiety and depression scores between LFD and control groups in the included studies. Eswaran et al. (23) demonstrated that LFDs could alleviate the symptoms of anxiety but not have any effects on depression. The other study conducted by Bohn et al. (22) showed that LFDs had no effect on depression in patients with IBS. At present, a limited number (only two papers included in this study) of studies cannot come to a definite conclusion on this proposition, and further studies are needed to put more focus on the effect of LFDs on improving the anxiety and depression statuses of IBS patients.

Quality assessment of the RCTs yielded high risk in the blinding process of one RCT (24) and in the outcome assessment process of another two RCTs (15, 23), although the overall risk of bias was relatively low. We used the GRADE methodology (53) to evaluate the quality of the evidence, which is the most widely accepted approach. Eventually, it was found that the evidence supporting the significant effects of LFDs on IBS symptoms was relatively reliable. Generally, the blinding of patients to the LFD can be challenging (52). Many IBS patients are aware of the concept of an LFD, and information on this diet is freely available. An IBS patient can easily deduce which diet they have been allocated to if they participate in an RCT. Only one paper (21) that was included had assessed blinding to the diets by asking participants to identify the diet that they had been allocated to prove the success of the blinding process. Therefore, adhering to a diet regime that is considered “healthy” might reduce anxiety and subsequently alleviate IBS symptoms; thus creating a placebo response. Most studies [except the three studies (15, 19, 23) that had only recruited IBS-D patients] did not address differences in responses to dietary interventions in IBS subgroups, making it difficult to demonstrate a difference in the response rates and other outcomes among IBS subtypes. However, according to the subgroup analysis, IBS-D patients seemed to get more benefits from an LFD in improving their bowel habits.

As reported, FODMAPs have important physiological effects: they increase stool bulk, enhance calcium absorption, modulate immune function, and decrease the levels of serum cholesterol, triacylglycerols, and phospholipids (48). Because of the effects mentioned above, many potential limitations and concerns about LFDs have been raised (21, 51) such as nutritional adequacy, cost, and difficulty in teaching, learning, and continuing the diet. Although a relatively short-term (<6 weeks) LFD was generally well-tolerated, with adverse events rarely reported (16, 26), the pooled mean difference of BMI was not statistically significant between LFD and control groups according to our study. The effects, both positive and negative, of a long-term LFD on IBS still need to be assessed by expanding the sample quantity and extending the time of intervention (52). Therefore, a minimum length of 6 months has been recommended to establish long-term efficacy (53).

This research has significant strengths. Firstly, on the basis of the previous meta-analyses (27, 30) on this topic, we have included new high-quality RCTs that were conducted recently after comprehensive retrieval and strict screening, increasing the total population and making the results more credible. Secondly, the risk of bias of every single trial was evaluated strictly according to the standards of Cochrane Risk of Bias Tool, and GRADE was used to evaluate the quality of evidence for each outcome. Although the study focused on the improvement of the overall symptoms of IBS, it also crucially analyzed the effects on stool output, quality of life, and anxiety and depression status. To our knowledge, this is the first meta-analysis to comprehensively evaluate the effects of an LFD on IBS symptoms from a multi-perspective. However, there are limitations to this systematic review as well. Firstly, the sample size of the participants involved is small. Additionally, most studies did not address differences in response to dietary interventions among IBS subgroups, which may exaggerate or minimize the effect of LFDs on specific subtypes of IBS. However, as shown in the pooled data, an LFD may be more effective in patients with IBS-D than those with constipation as a major symptom. Finally, different studies did not use a unified evaluation scale, such as IBS-SSS, to evaluate the overall symptoms of IBS. Different definitions of IBS symptom improvement may limit the reported benefit of LFDs in IBS patients and lead to a certain degree of heterogeneity among different studies.

In conclusion, this systematic review and meta-analysis provide a moderate quality of evidence for supporting the efficacy of an LFD in the improvement of global symptoms and bowel habits of adult IBS patients. The improvement in bowel habits seems to be more pronounced in IBS-D patients. Recommending adult IBS patients, especially those with IBS-D, to try an LFD with professional advice from health care professionals is worth promoting.

## Data Availability Statement

The raw data supporting the conclusions of this article will be made available by the authors, without undue reservation.

## Author Contributions

JW came up with the idea of the study. JW and LZ designed the research. JW, PY, LZ, and XH conducted the research, analyzed the data, and performed the statistical analysis. JW and PY wrote initial version. LZ and XH provided critical input. All authors had equal responsibility for the final content of the paper, read, and agreed to the published version of the manuscript.

## Conflict of Interest

The authors declare that the research was conducted in the absence of any commercial or financial relationships that could be construed as a potential conflict of interest.

## Publisher's Note

All claims expressed in this article are solely those of the authors and do not necessarily represent those of their affiliated organizations, or those of the publisher, the editors and the reviewers. Any product that may be evaluated in this article, or claim that may be made by its manufacturer, is not guaranteed or endorsed by the publisher.
