# Supplementary material for: A Low-FODMAP Diet Improves the Global Symptoms and Bowel Habits of Adult IBS Patients: A Systematic Review and Meta-Analysis
Source: Front Nutr. 2021 Aug 19;8:683191. doi: 10.3389/fnut.2021.683191 (PMC8417072; doi:10.3389/fnut.2021.683191)
Supplement: Supplementary file 1 [file Data_Sheet_1.pdf]

Supplementary Material

1 Supplementary figures

Study or Subgroup	LFD Mean	LFD SD	LFD Total	ND Mean	ND SD	ND Total	Weight	Mean Difference IV, Random, 95% CI
Böhn 2015	42.2	32.6	33	37.6	26.9	34	9.8%	4.60 [-9.73, 18.93]
McIntosh 2017	19.9	18.4	18	41.5	26.1	19	9.6%	-21.60 [-36.09, -7.11]
Staudacher 2017	30	27	51	44	29	53	15.0%	-14.00 [-24.76, -3.24]
Wilson 2020	30.1	5.1	22	42.7	5	23	40.4%	-12.60 [-15.55, -9.65]
Zahedi 2018	16.13	13.13	50	25.88	21.11	51	25.2%	-9.75 [-16.59, -2.91]
**Total (95% CI)**			**174**			**180**	**100.0%**	**-11.27 [-16.32, -6.23]**

Heterogeneity: Tau² = 14.12; Chi² = 7.54, df = 4 (P = 0.11); I² = 47%
Test for overall effect: Z = 4.38 (P < 0.0001)

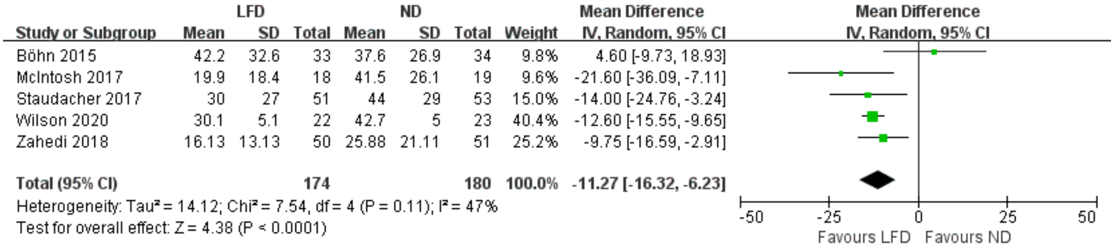

Supplementary Figure 1: Pooled mean difference for pain intensity

Study or Subgroup	LFD Mean	LFD SD	LFD Total	ND Mean	ND SD	ND Total	Weight	Mean Difference IV, Random, 95% CI
Böhn 2015	43.6	30.6	33	37.8	26.5	34	14.7%	5.80 [-7.92, 19.52]
McIntosh 2017	41.9	27.7	18	66.8	28.3	19	10.5%	-24.90 [-42.95, -6.85]
Staudacher 2017	30	27	51	44	29	53	18.6%	-14.00 [-24.76, -3.24]
Wilson 2020	30.1	5.1	22	42.7	5	23	30.4%	-12.60 [-15.55, -9.65]
Zahedi 2018	13.25	14.21	50	16.75	17.74	51	25.7%	-3.50 [-9.76, 2.76]
**Total (95% CI)**			**174**			**180**	**100.0%**	**-9.11 [-16.26, -1.96]**

Heterogeneity: Tau² = 41.51; Chi² = 14.95, df = 4 (P = 0.005); I² = 73%
Test for overall effect: Z = 2.50 (P = 0.01)

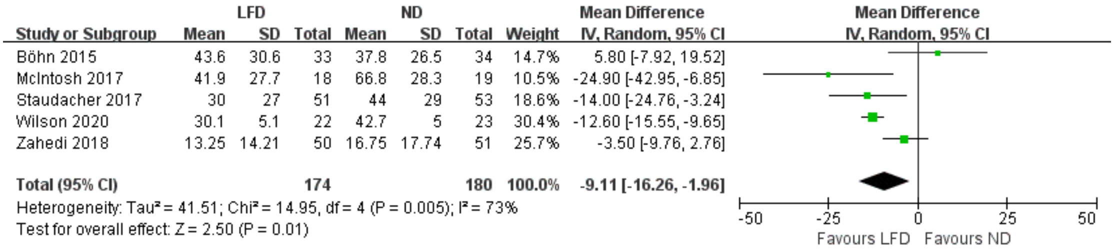

Supplementary Figure 2: Pooled mean difference for pain frequency

Study or Subgroup	LFD Mean	LFD SD	LFD Total	ND Mean	ND SD	ND Total	Weight	Mean Difference IV, Random, 95% CI
Böhn 2015	55.9	31	33	58.6	24.3	34	3.1%	-2.70 [-16.06, 10.66]
McIntosh 2017	57.3	14.2	18	65.9	23.6	19	3.5%	-8.60 [-21.08, 3.88]
Staudacher 2017	40	20	51	47	21	53	8.8%	-7.00 [-14.88, 0.88]
Wilson 2020	41.1	4.7	22	53.8	4.6	23	74.1%	-12.70 [-15.42, -9.98]
Zahedi 2018	20.13	18.55	50	31.25	18.56	51	10.5%	-11.12 [-18.36, -3.88]
**Total (95% CI)**			**174**			**180**	**100.0%**	**-11.58 [-13.92, -9.24]**

Heterogeneity: Tau² = 0.00; Chi² = 3.88, df = 4 (P = 0.42); I² = 0%
Test for overall effect: Z = 9.70 (P < 0.00001)

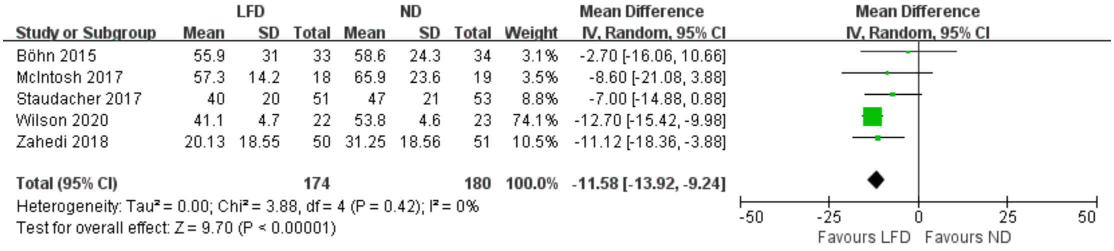

Supplementary Figure 3: Pooled mean difference for interference on life in general

Study or Subgroup	LFD Mean	LFD SD	LFD Total	ND Mean	ND SD	ND Total	Weight	Mean Difference IV, Random, 95% CI
Böhn 2015	58.5	31.2	33	53.4	25.3	34	6.5%	5.10 [-8.52, 18.72]
McIntosh 2017	56.2	23	18	67.6	26.9	19	4.8%	-11.40 [-27.50, 4.70]
Staudacher 2017	42	23	51	53	17	53	16.9%	-11.00 [-18.80, -3.20]
Wilson 2020	44.6	5.1	22	55.1	5	23	50.0%	-10.50 [-13.45, -7.55]
Zahedi 2018	31.75	17.37	50	39.25	16.23	51	21.8%	-7.50 [-14.06, -0.94]
**Total (95% CI)**			**174**			**180**	**100.0%**	**-8.95 [-12.60, -5.31]**

Heterogeneity: Tau² = 4.65; Chi² = 5.39, df = 4 (P = 0.25); I² = 26%
Test for overall effect: Z = 4.82 (P < 0.00001)

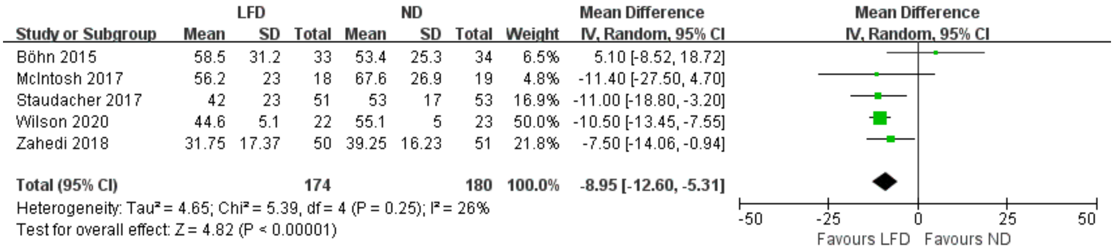

Supplementary Figure 4: Pooled mean difference for dissatisfaction of bowel habit

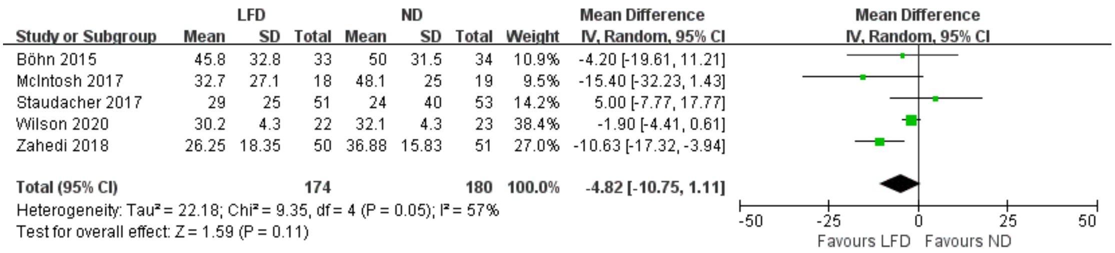

Supplementary Figure 5: Pooled mean difference for abdominal distension

2 Supplementary Tables

Supplementary Table 1： Subgroup analysis of the outcomes in this study.

Outcome or subgroup	Studies	Participants	Effect Estimate (95% confidence interval)	Heterogeneity: $I^2(\%)$	Test for subgroup differences: I^2 (%)
**1.Global symptom improvement rate**	**7**	**420**	**1.54 [1.18, 2.00]**	**38**	
Treatment duration					0
3 weeks	2	70	1.84 [0.61, 5.60]	78	
4 weeks	5	350	1.52 [1.15, 2.02]	30	
FODMAP level in control diet					**63**
High FODMAP	1	40	3.25 [1.28, 8.27]	Not applicable	
Normal FODMAP	6	380	1.45 [1.14, 1.84]	25	
Definition of clinically meaningful improvement					0
A reduction≥50 in IBS-SSS	2	115	1.75 [0.59, 5.18]	77	
A reduction≥10 in VAS	1	30	1.19 [0.75, 1.88]	Not applicable	
Had adequate relief over the past 7 days	4	275	1.67 [1.27, 2.20]	10	
IBS subtype					0
IBS-D	1	85	1.27 [0.80, 2.03]	Not applicable	
All subtypes	6	335	1.62 [1.18, 2.22]	45	
**2. IBS-QOL**	**5**	**484**	**2.77 [-2.00, 7.55]**	**62**	
Treatment duration					0
4 weeks	4	383	3.54 [-1.86, 8.94]	66	

6 weeks	1	101	-1.22 [-9.55, 7.11]	Not applicable	
IBS subtype					0
IBS-D	2	185	4.88 [-5.96, 15.73]	81	
All subtypes	3	299	0.72 [-3.23, 4.67]	0	
**3. IBS-SSS**	**5**	**354**	**-37.72 [-53.97, -21.46]**	**40**	
Treatment duration					21.6
3 weeks	1	37	-82.00 [-140.87, -23.13]	Not applicable	
4 weeks	3	216	-30.98 [-54.32, -7.64]	47	
6 weeks	1	101	-41.75 [-64.37, -19.13]	Not applicable	
FODMAP level in control diet					**59**
High FODMAP	1	37	-82.00 [-140.87, -23.13]	Not applicable	
Normal FODMAP	4	317	-34.50 [-43.86, -25.14]	29	
IBS subtypes					0
IBS-D	1	101	-41.75 [-64.37, -19.13]	Not applicable	
All subtypes	4	253	-34.46 [-44.59, -24.33]	53	
**4.Stool consistency**	**6**	**434**	**-0.25 [-0.44, -0.06]**	**19**	
Treatment duration					0
4 weeks	5	333	-0.21 [-0.49, 0.06]	32	
6 weeks	1	101	-0.31 [-0.54, -0.08]	19	
IBS subtype					7.4
IBS-D	2	183	-0.34 [-0.55, -0.14]	0	
All subtypes	4	251	-0.14 [-0.46, 0.19]	35	
**5.Stool frequency**	**6**	**434**	**-0.28 [-0.57, 0.01]**	**83.9**	
Treatment duration					**83.9**
4 weeks	5	333	-0.18 [-0.44, 0.09]	48	
6 weeks	1	101	-0.69 [-1.00, -0.38]	Not applicable	
IBS subtype					**84.6**
IBS-D	2	183	-0.67 [-0.96, -0.38]	0	
All subtypes	4	251	-0.14 [-0.43, 0.15]	57	
